# The Role of the Unitary Prevention Delegates in the Participative Management of Occupational Risk Prevention and Its Impact on Occupational Accidents in the Spanish Working Environment

**DOI:** 10.3390/ijerph17165678

**Published:** 2020-08-06

**Authors:** Raúl Payá Castiblanque

**Affiliations:** Department of Sociology and Social Anthropology, University of Valencia, 46022 Valencia, Spain; raul.paya@uv.es; Tel.: +34-650-157-401

**Keywords:** unitary prevention delegates, preventive management, workers’ participation, cultural activation, accidents at work

## Abstract

The aim of this research was to study the impact of the unitary prevention delegates (UPDs) on the Spanish working environment. To this end, a cross-sectional study was carried out using microdata from the National Survey on Health and Safety Management in Companies (ENGE-2009) with a sample of 5147 work centres. To measure the relationship between the presence of UPD in workplaces with preventive management indicators and damage to health, individual and multiple logistic regression models were carried out, calculating the crude (cOR) and adjusted (aOR) odds ratios by sociodemographic covariates, with their corresponding 95% confidence intervals (95% CI). Ambivalent results were obtained. On the one hand, a positive impact of the UPDs was found, in the management of prevention showing a higher probability of prevention plans being carried out (aOR = 3.97; 95% CI: 3.26–4.83), risk assessments (aOR = 5.96; 95% CI: 4.44–8.01) and preventive actions were planned (aOR = 3.01; 95% CI: 2.55–3.56), as well as 1.56 times less likely to register minor occupational accidents (aOR = 0.64; 95% CI: 0.53–0.76). On the other hand, the presence of the UPDs did not promote the activation of a participatory culture and did not reduce the probability of suffering serious and fatal accidents at work. In conclusion, UPDs need to activate workers’ participation to improve results.

## 1. Introduction

With the approval of Law 31/1995 on the Prevention of Occupational Risks, in Spanish “*Ley de Prevención de Riesgos Laborales*” (LPRL) [1], and its subsequent development with the Regulations on Prevention Services (Royal Decree 39/1997) [2], the Spanish state ratified and transposed, although five years late, the European Union Framework Directive 89/391-CEE [3], inaugurating a new stage in the prevention of risks in the workplace and the promotion of occupational health. The regulatory obligations in the field of prevention stipulated by the LPRL had a positive impact on reducing the incidence rates for accidents at work [4]. Specifically, official records [5] showed a sustained reduction in accidents, going from an incidence rate of 7437.4 accidents per 100,000 workers in 2000 to an indecency rate of 2948.8 in 2012, which meant a reduction in the rate of variation of occupational accidents of 60.35% [6]. This positive evolution was interrupted by the beginning of the financial crisis and the stagnation of the global economy in 2008 (*the great recession*) as austerity policies were adopted (*the great aggression*) imposed by the Troika (formed by the European Commission), the European Central Bank and the International Monetary Fund) based on a political exchange of “neoliberal intergovernmentalism” that forced the member states of the European Union in economic difficulties, especially the countries of the south, to deregulate the labour market and labour relations [7,8,9] with the “conditionality” of obtaining financial aid and bank bailouts [10]. This aggression has led to a *great regression* in employment and work conditions, increasing the precariousness of work [11]. 

In the Spanish case, social deregulation occurred through the approval of Royal Decree-Law 3/2012, which increased contractual precariousness, devaluation of salaries and weakening of social protection systems [12], with heterogeneous and controversial effects on the population’s health [13,14,15]. Precisely in 2012, the turning point in the incidence rate for occupational accidents was reached, and since then it has increased by 15.59% to 3408.7 accidents per 100,000 workers by the end of 2018 [5], describing a trend that is radically opposed to that of the European Union, whose standardised incidence rate is 1666.34 [16], with Spain, along with other southern countries (Portugal and France), being the European country with the most occupational accidents. Numerous studies have shown that temporary contracts and high turnover are determining factors in the increase in occupational accidents [17,18,19,20]. In particular, temporary workers in Spain were 2.94 times more likely to have non-fatal accidents at work in 2000–2001 and 2.54 times more likely to suffer a fatal accident than those hired for an indefinite period [19], while other studies found that workers with less than one year’s seniority in the workplace were 3.09 times more likely to suffer an accident at work than those who had been in the workplace for more than three years [20]. The 2012 labour reform boosted temporary contracts, making Spain the European Union country with the highest rate of temporary contracts, with 26.9% in 2018 [21], with contracts of increasingly shorter duration (50.6 days on average in 2017) [6]. Thus, based on previous studies, it is possible to link the increase in job insecurity with the evolution of workplace accidents. In fact, some researchers consider the increase in workplace accidents to be a kind of “toll” or “collateral damage” for overcoming economic crises [22].

After analysing the labour market determinants that have affected the increase in occupational accidents, the factors in occupational risk prevention management that influence the evolution of the incidence rates are analysed below. On the one hand, it is known that companies with high standards of preventive management (risk assessment, preventive planning, training and information on risks) have lower accident rates than companies not concerned with occupational health and safety [23,24,25,26]. However, the economic crisis has also produced a relaxation of companies’ compliance with preventive standards, focusing more on improving productivity and financial problems than on occupational health and safety [27,28,29]. It has also been shown that direct worker participation in preventive management improves absenteeism and accident rates [30,31,32]. These studies have shown that companies with a participatory culture in which workers are involved in identifying occupational risks and designing and implementing prevention measures (active participation) have fewer occupational accidents than companies that do not allow workers to participate or simply inform them of the risks to which they are exposed (passive participation). However, again, the combined effect of the financial crisis and authoritarian organisational cultures reduces the possibilities for active participation of workers both in Europe in general [33] and in the Spanish system of industrial relations in particular [34,35].

In this context, trade union representatives have had, and still have, a relevant role in defending workers’ health and safety throughout history [36,37]. In this sense, there is scientific evidence of the positive indirect and direct impact of the presence of unionised workers’ representatives specialising in occupational health (prevention delegates) in work centres, with the improvement of working conditions and risk prevention [38,39,40]. Specifically, union prevention delegates have an indirect impact on occupational health because, in those work centres where they are present, they are able to pressure management to comply with their legal obligations, manage risk prevention by carrying out preventive evaluations and planning [39,40] and activate direct worker participation in preventive management processes [41,42]. Thus, as mentioned above, greater management and participation reduces the incidence rates of occupational accidents. and, therefore, trade union representatives with the activation of participatory management influence occupational accidents indirectly. The direct impact of union representation would be given by the direct relationship between their struggle and participation in the improvement of working conditions and the reduction of occupational accidents [43,44]. In this regard, other studies have focused on analysing the determinants of the effectiveness of union prevention delegates [45,46,47]. These include the role of business leadership in promoting participatory management to facilitate the work of prevention delegates [48,49,50,51], a labour inspectorate that involves key interlocutors in prevention management systems [52,53] and the fact that prevention delegates are unionised and have the support of the unions [54,55,56,57].

With reference to the latter determinant, a recent comparative qualitative study between the countries of the European Union developed by Walters and Wadsworth in 2020 [33] showed that non-unionised systems of unitary representation are becoming increasingly widespread and company managers tend to appropriate such representation, displacing and marginalising union representatives and endangering the fragile balance between capital and labour. This situation leads to less effectiveness of prevention delegates when they are part of unitary representation than when they are members of a union [41,42]. Spain is a clear example of a labour relations system based on worker representation through unitary prevention delegates [58,59]. Comparative European studies show that, while Anglo-Saxon systems of labour relations are characterised by voluntary systems with decentralised self-regulation and based on a high level of trade union pluralism derived from the single channel (trade union) of representation in the workplace, the countries of the Mediterranean are institutionalised countries with a high level of state intervention, and, therefore, the system of representation of interests has a double channel (trade union and unitary), but the system revolves around the electoral route (unitary representation), which allows the representation of interests to be expanded for non-unionised workers. Nevertheless, it produces a free rider effect that discourages union membership and weakens the associative power of the unions [60,61]. In fact, in Spain, representation doubles the number of union members, with a union affiliation rate of 18.9%, while elected personnel delegates (unitary representation) increase representation to 53% of the active population [61]. The different systems of general representation influence the system of occupational health and safety representation, so that in Anglo-Saxon countries prevention delegates are elected by the unions themselves and from among union members, while in Mediterranean countries prevention delegates are elected by direct vote of the workers or from the unitary representation (personnel delegates) [58]. In the case of Spain, Article 35.2 of the LPRL [1] states that “the Prevention Delegates shall be appointed by and from among the staff representatives”, that is, a system of unitary prevention delegates is clearly established. In conclusion, the unitary prevention delegates are those workers’ representatives with specific functions in the field of occupational safety and health in the workplace who have been elected from among the unitary representatives (elected by the workers), and, therefore, this specialised risk prevention system is characterised by the fact that it is built on workers elected as personnel delegates and not on trade union representatives. Thus, the lack of direct involvement of trade unions in this specialised figure of representation of interests in occupational health and safety has resulted in a growing scientific concern about the effectiveness of the system.

**Figure 1:**
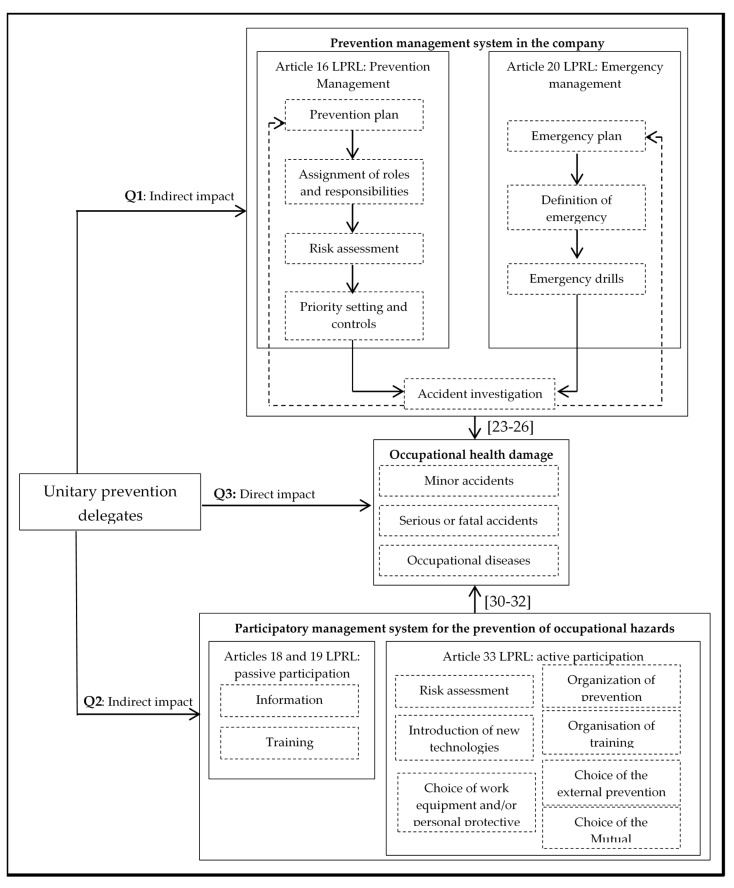
Participatory management system for the prevention of occupational risks. **Q1**, first research question; **Q2**, second research question; **Q3**, third research question; LPRL, Law of 31/1995 on the Prevention of Occupational Risks; [23,24,25,26], Number of articles in the bibliographical references that have demonstrated a positive impact of preventive management on accidents; [30,31,32], number of articles in the bibliographical references that have demonstrated a positive impact of workers’ participation on accidents.

From the analysis of the previous studies, it can be concluded that, while a positive direct and indirect impact of the unionised prevention delegates coming from Anglo-Saxon labour relations systems has been found, there is no comparable scientific evidence on the impact of unitary prevention delegates promoted in the Mediterranean countries. In addition, that there is a strong concern about the effectiveness of these unitary representation systems given their growing expansion in the rest of the European Union countries. Therefore, the aim of this research was to study the direct and indirect impact of the unitary prevention delegates in the Spanish work environment, answering for this purpose the following research questions (Figure 1): (Q1) In the work centres where there are unitary prevention delegates, will there be better standards of preventive management and will the workers’ representatives be able to pressure the managers to comply with the LPRL (indirect impact)? (Q2) Will these representatives succeed in activating a participatory culture in which workers participate in risk prevention management (indirect impact)? (Q3) Will the unitary prevention delegates achieve a lower number of occupational accidents (direct impact)?

It should be recalled, as shown in Figure 1, that the LPRL establishes both the business obligation to manage the prevention of occupational risks and the right of workers to participate in such management. Specifically, with regard to management elements, on the one hand, Article 16 of the LPRL determines that companies: (a) must draw up a prevention plan in which they assign functions and responsibilities in the area of prevention; (b) must identify the dangers through risk assessment; (c) must plan preventive action based on the risks identified in the assessment phase and establish priorities and controls on the effectiveness of the planned preventive activities; and (d) must investigate the causes of accidents at work by generating feedback on the preventive process. On the other hand, Article 20 of the LPRL, in reference to emergency measures, states that: (a) a self-protection plan must be drawn up; (b) emergency measures must be defined; and (c) practices derived from the emergency plan must be carried out. Finally, with reference to the participation of workers in preventive management, Articles 18 and 19 of the LPRL determine that all workers must be informed and trained about the risks of their jobs and the emergency measures (passive participation), as well as actively participate (Article 33 of the LPRL) in: (a) drawing up risk assessments; (b) introducing new technologies; (c) choosing work equipment and personal protective equipment; (d) organising prevention at the workplace; (e) organising training; and (f) and choosing ways of integrating prevention into the company (prevention services and mutual accident insurance). Thus, the first two questions of this research (Q1 and Q2) focus on identifying whether work centres with unitary prevention delegates achieve compliance with the law by activating participatory prevention management (indirect impact). The direct impact (Q3) of the representatives focuses on analysing the final effectiveness of the system in terms of accidents at work.

## 2. Materials and Methods 

### 2.1. Source and Sample

Quantitative research that seeks to study the relationships between worker representation systems and occupational health management indicators is rare because of the difficulty of obtaining national surveys of quality working conditions that provide variables for analysis [33]. Therefore, despite being little known and used, the National Survey on Health and Safety Management in Companies (ENGE) [62] carried out in 2009 by the Spanish National Institute of Health and Safety at Work (INSHT), provides unique conditions for conducting a cross-sectional study to analyse the objectives of this research. There are several reasons for this choice. Firstly, the survey ENGE has a population of 5147 companies of all economic activities and staff sizes, belonging to the entire national territory (with the exception of Ceuta and Melilla) and having at least one worker registered with Social Security. In addition, the selection of the sample was carried out based on a universe of 1,120,276 companies, stratified according to their economic activity and staff size, carrying out a minimum of 180 interviews in each Autonomous Community and proportionally distributing the remaining ones based on the number of quotation centres in each one of them. Therefore, a representative sample of the Spanish productive fabric was obtained which allowed reliable statistical analyses to be carried out, since, for a confidence level of 95.5% (two sigmas) and P = Q, the error for the sample as a whole was 1.39%. Secondly, the fieldwork was carried out from 22 January to 15 May 2009, and the entrepreneurs or those responsible for prevention were interviewed in person at the companies. The period of time included coincides with the hardest moments of the economic crisis and, therefore, taking into consideration the described context, it is an ideal period to study the impact of the unitary prevention delegates on the preventive management system and to prevent companies from relaxing prevention standards. Thirdly, the questionnaire applied has 57 questions, structured in nine information blocks, of which the following should be highlighted for the purposes of this research: Block F entitled “Activities for the prevention of occupational risks”, Block D called “Participation bodies” and Block H called “Damage to health”. It is worth mentioning that the survey not only has indicators of all the elements necessary to develop the research, but that the questions asked are developed based on the articles of the LPRL described in the Introduction, which has allowed a precise study of legal compliance in the dimensions of both preventive management and worker participation.

### 2.2. Independent Variable (Presence or Absence of Prevention Delegates)

This is a dichotomous variable constructed on the basis of Question 17 belonging to Block D of the ENGE survey, which asks about the presence or absence of unitary prevention delegates in the workplace. Table 1 shows the distribution of the sample of worker representation according to size and sector of activity. As can be seen, there is a positive correlation between the size and the presence of prevention delegates, since, while in companies with fewer than 10 workers the number of work centres with prevention delegates is 19.5%, in the largest companies all have representatives. In addition, those sectors with a longer tradition of labour relations (construction and industry) also have higher levels of representation. These results are in line with previous studies [9,46], which would confirm that the sample is representative of the Spanish productive fabric.

### 2.3. Dependent Variables

The dependent variables are grouped according to the three blocks mentioned in the analysis of the sources. Specifically, Block F of the ENGE survey provides nine indicators related to activities for the prevention of occupational risks linked to the first research question (Q1). The first indicator was extracted from Question 32, which stated that “In this work centre, has the evaluation of the risks to the safety and health of the workers been carried out”, with three possible answers (Yes, it has been carried out/No, it has not been carried out/It is planned). For the construction of the variable, the response alternatives were dichotomised (Yes, carried out/No, not carried out or planned). The other eight management indicators were extracted from Question 38 in which the person interviewed had to indicate what other activities related to the prevention of occupational risks had been or were being carried out in the workplace, the activities in question being: drawing up the prevention plan; assigning those in charge the obligation to include prevention in all the decisions they take; planning preventive activity; establishing priorities and controls effectiveness of preventive activities drawing up a self-protection plan; defining emergency measures; practices derived from the emergency plan (evacuation drills, etc.); and investigation of accidents at work The eight indicators were converted into dummy variables in the same way as the risk assessment dimension (Yes, done/No, not done or planned).

With reference to the indicators of direct worker participation in risk prevention management used to answer the second research question (Q2), nine indicators were selected. On the one hand, two questions from Block F of the ENGE survey were used to measure passive participation: Question 34, “Are workers informed of the results of the risk assessment that affects their job or function?”, and Question 41, “During the last two years, have any training activities or activities on occupational safety and health been carried out at this workplace?” On the other hand, to measure the degree of active participation, Question 22 of Block D was used to analyse the participation bodies. The statement of the question was: “In this workplace, on which of the following aspects related to health and safety at work are workers consulted”, with seven indicators or aspects to be measured: risk assessment; introduction of new technologies; choice of work equipment and/or personal protection equipment; organisation of prevention of occupational risks; organisation of training; choice of other people’s prevention service; and choice of the mutual insurance company covering occupational accidents and diseases. Both indicators of passive participation and the seven of active participation had two response alternatives (Yes/No), and no transformation of the variables was necessary.

Finally, to measure the direct impact of the unitary prevention delegates on the levels of accidents at work (Q3), Question 49 of Block H was used, which stated: “In the last two years, indicate the accidents at work (excluding “in itinere” accidents) and occupational diseases that occurred in the workplace”. In this regard, of the 5147 companies consulted, 1064 (20.47%) stated that they had had minor accidents in the last two years, 61 (1.2%) serious accidents, 8 (0.2%) fatal accidents and 70 (1.4%) occupational diseases. The reduced frequency of fatal accidents did not allow such stratified statistical analysis, so three dependent indicators were considered: minor accidents; serious and/or fatal accidents; and occupational diseases. Table 2 shows the relationship of all variables included in this investigation.

### 2.4. Model Adjustment Covariates

As discussed in the Introduction, there are sociodemographic factors that would explain both the levels of preventive management and worker participation [46,47] and the incidence rates of occupational accidents [17,18,19,20]. Therefore, to avoid finding spurious relationships between the presence or absence of unitary prevention delegates in the work centres (independent variable) with the indicators of preventive management, worker participation and occupational accidents (dependent variables), several sociodemographic variables were included in the statistical models. On the one hand, the company size and the activity sector are included in the models (Table 1). On the other hand, the ENGE survey has many elements to measure the working conditions, being again a relevant factor to be able to compare the degree of impact of the prevention delegates with the indicators of precariousness at work on the preventive management and work accidents. The characteristics and working conditions included in the statistical models are the type of contract for own staff (permanent/temporary); work on the premises of external staff (self-employed/temporary/subcontracted); the type of working day (morning and afternoon/continuous morning/continuous afternoon/continuous night/morning and afternoon shifts/morning, afternoon and night shifts); sex (male/female); nationality (nationals/foreigners); and age (between 16 and 19 years/between 20 and 24 years/between 25 and 54 years/between 55 and 65 years). To include the twenty indicators grouped into the six dimensions in the statistical models, several operations were carried out. Firstly, the survey only provides the absolute number of workers assigned to each indicator and, therefore, the percentage of workers over the total number of people working in the company was calculated for each of the twenty indicators. Secondly, after transforming the absolute data into a percentage, the twenty indicators were dichotomised by their average. Thus, dummy variables were constructed by dividing the companies between those below the average and those below in each of the indicators (Table 2). Table A1 shows the descriptive statistics of the sample.

### 2.5. Statistical Analysis

To measure the relationship between the presence of the unitary prevention delegates in the work centres with the indicators of preventive management, direct participation of the workers and damage to health, two statistical analyses were carried out. First, the prevalences of the 21 dependent variables were calculated according to the presence or absence of the prevention delegates. Secondly, through individual and multiple logistic regression models, crude (cOR) and adjusted (aOR) odds ratios were calculated for the socio-demographic covariates, with their corresponding 95% confidence intervals (95% CI), establishing as a reference category for the dependent variables the absence of preventive management, partitioning or damage to health. All calculations were performed with SPSS version 26 statistical software (IBM Corp, Armonk, NY, USA).

### 2.6. Ethical Considerations 

The research uses databases from the Spanish National Institute of Safety and Hygiene in the Workplace, considered a reliable source of data, which acts with ethical procedures and the data downloaded are anonymous. This study was approved by the Human Research Ethics Committee of the University of Valencia’s Ethics in Experimentation Commission (code UV-INV_ETICA-1392093).

## 3. Results

### 3.1. Impact on Preventive Management

The results obtained (Table 3) show how in work centres with unitary prevention delegates there is greater risk prevention and emergency management. In reference to preventive management, in places where there are prevention delegates, risk assessments were carried out in 94.3% of the workplaces, while those workplaces where there are no representatives were only assessed in 71.8% of the cases, which means that, in those workplaces that have prevention delegates, they were 5.96 times more likely to have a risk assessment (aOR = 5.96; 95% CI: 4.44–8.01), as well as more likely to have a prevention plan (aOR = 3.97; 95% CI: 3.26–4.83), assign prevention roles and responsibilities (aOR = 2.15; 95% CI: 1.86–2.50), plan prevention actions (aOR = 3.01; 95% CI: 2.55–3.56) and establish controls and priorities (aOR = 2.10; 95% CI: 1.82–2.44). With regard to emergency management, it was also found that work centres with unitary prevention delegates were more likely to have emergency plans (aOR = 2.06; 95% CI: 1.77–2.38), as well as more likely to have defined emergency measures (aOR = 1.58; 95% CI: 1.36–1.83) and to have carried out drills (aOR = 2.16; 95% CI: 1.85–2.52). Likewise, the presence of prevention delegates also guarantees the feedback of the occupational health and safety management system, since occupational accidents are investigated more frequently (aOR = 1.50; 95% CI: 1.29–1.74) so that faults in the system can subsequently be found and corrected.

### 3.2. Impact on the Activation of Participatory Management

In reference to the impact of the unitary prevention delegates on the activation of worker participation in occupational safety and health, the results are ambivalent (Table 4). On the one hand, they had a positive impact on the activation of passive participation to the extent that the centres with unitary representation were 1.38 times more likely to have informed workers about the risks of the workplace (aOR = 1.38; 95% CI: 1.09–1.74) and 3.51 times more likely to have been trained about the risks to which they are exposed (aOR = 3.51; 95% CI: 2.97–4.15). On the other hand, the presence of delegates affected negatively all the indicators of active participation to the extent that workers were less consulted about the identification of risks in the evaluation process (aOR = 0.47; 95% CI: 0.40–0.55), as well as less involved in the choice of new technologies (aOR = 0.57; 95% CI: 0.49–0.67) or work and personal protection equipment (aOR = 0.71; 95% CI: 0.61–0.82). Likewise, workers with prevention delegates were less likely to participate in the organisation of prevention (aOR = 0.61; 95% CI: 0.52–0.71) or training (aOR = 0.49; 95% CI: 0.42–0.57) and the choice of the third-party prevention service (aOR = 0.71; 95% CI: 0.58–0.86) or mutual insurance company for occupational accidents (aOR = 0.57; 95% CI: 0.48–0.69).

### 3.3. Impact on Occupational Health Damage

The direct impact of the prevention delegates on occupational health hazards is shown in Table 5. The results show that, in the work centres that had unitary prevention delegates, there was 1.56 times lower probability of minor work accidents occurring (aOR = 0.64; 95% CI: 0.53–0.76; *p*-value = 0.000) and this relationship was considered significant, as the *p* value was less than 0.05. Likewise, it was observed that, in the companies that presented higher than average percentages of men (aOR = 1.29; 95% CI: 1.11–1.51; *p*-value = 0.001), over 65 years of age (aOR = 3.09; 95% CI: 1.95–4.80; *p*-value = 0.000), temporarily hired (aOR = 1.35; 95% CI: 1.04–1.75; *p*-value = 0.027) or from outside the company (aOR = 5.76; 95% CI: 1.99–16.65; *p*-value = 0.000 for freelancers; aOR = 2.91; 95% CI: 1.91–4.45; *p*-value = 0.001 for subcontractors), who work rotating shifts in the morning, afternoon and night (aOR = 5.26; 95% CI: 3.35–5.19; *p*-value = 0.000), in companies with 250 to 499 workers (aOR = 5.66; 95% CI: 5.43–30.54; *p*-value = 0.001) and in the construction sector (aOR = 1.90; 95% CI: 1.30–2.75; *p*-value = 0.000), were those with the highest probability of reporting minor occupational accidents, these relationships being significant.

Despite the fact that the unitary prevention delegates showed a positive impact on the reduction of light occupational accidents, they did not have the same effect on serious or fatal accidents and occupational diseases, as the results found do not identify statistically significant relationships (*p*-value = 0.785 for serious and fatal accidents; *p*-value = 0.163 for occupational diseases), the factors associated with precariousness of work being the predictors of the negative impact on more serious health damage. Specifically, it was observed that those companies that abuse subcontracting have more serious and fatal accidents (aOR = 3.07; 95% CI: 1.36–6.93; *p*-value = 0.007), while working rotating morning, afternoon and night shifts has been associated with a higher probability of suffering from occupational diseases (aOR = 6.01; 95% CI: 1.21–14.71; *p*-value = 0.000).

**Figure 2:**
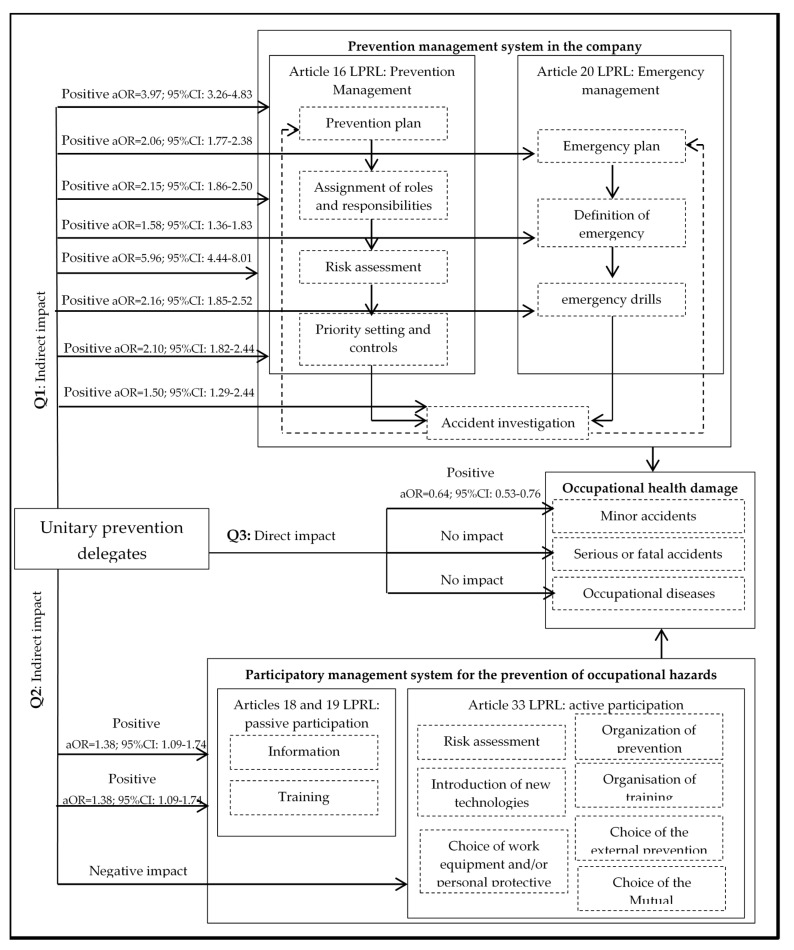
Results of the Participatory Management System for the Prevention of Occupational Risks. **Q1**, first research question; **Q2**, second research question; **Q3**, third research question; aOR, adjusted odds ratio; 95% CI, 95% confidence interval; LPRL, Law of 31/1995 on the Prevention of Occupational Risks.

If we return to the flow of the process of participatory preventive management (Figure 1) and use the results obtained from the statistical analyses carried out in Table 3, Table 4 and Table 5, it is possible to answer the three research questions. Thus, by organising the results obtained in the flow or process of participatory management (Figure 2), it can be seen that, in work centres that have unitary prevention delegates, the LPRL is complied with to a greater extent, since there are better standards of preventive management showing an indirect positive impact on work accidents (Q1). In addition, there is also a positive relationship with passive participation indicators (information and training); nevertheless, the impact is negative on the deeper levels of participation, showing little effectiveness with the activation of a participatory preventive culture, thus it does not have an indirect positive impact on occupational injuries (Q2). These ambivalent results translate into a moderate direct impact, as the presence of prevention delegates in the workplaces is related to a lower probability of referring minor occupational accidents, but they do not succeed in reducing the most serious damage to health (serious or fatal accidents and occupational diseases) (Q3).

### 3.4. Simplification and Summary of the Participatory Management Model

**Figure 3:**
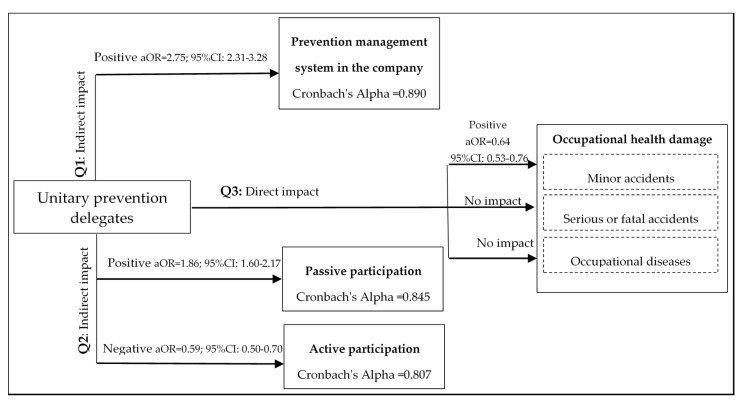
Simplification of the model and summary of results of the Participatory Management System for the Prevention of Occupational Risks. **Q1**, first research question; **Q2**, second research question; **Q3**, third research question; aOR, adjusted odds ratio; 95% CI, 95% confidence interval.

Although each indicator studied has its own purpose and, for this reason they have been analysed separately, to simplify and summarise the model in the four dimensions studied (management system for prevention in the company; passive participation of workers; active participation; and damage to occupational health), they were analysed together (Figure 3). In this sense, first, an analysis of the reliability of the indicators belonging to each dimension was carried out through the Cronbach’s Alpha test to verify that they belong to the same construction. As expected, the nine preventive management indicators showed a high internal consistency (Cronbach’s Alpha = 0.890), which resulted in values close to 1, showing high reliability of the measurement scale, as well as the two indicators with passive participation (Cronbach’s Alpha = 0.845) and the seven indicators of active participation (Cronbach’s Alpha = 0.807), while the damage to health showed low internal consistency (Cronbach’s Alpha = 0.191) and, therefore, could not be simplified. After the reliability analysis, the indicators for each dimension were added up. In this way, a scale of measurement was obtained for each dimension, which in the case of preventive management ranged from 0 (no indicator managed) to 9 (all indicators managed), while in passive participation it ranged from 0 (not reported or trained) to 2 (trained and reported) and in active participation from 0 (no participation indicator) to 7 (all participation indicators). After the summation, we proceeded to dichotomise the scales of the dimensions by their median: 4 for preventive management; 2 for passive participation; and 3 for active participation. Thus, the resulting dummy variables constructed were: preventive management (No management of prevention = 0 to 4/Prevention management = 5 to 9); passive participation (No participation = 0 to 1/Yes participation = 2); and active participation (No participation = 0 to 3/Yes participation = 4 to 7). Once the model was simplified, the adjusted logistic regressions were performed as the rest of the analyses performed in the present study. Thus, it can be summarised that the presence of unitary prevention delegates in the work centres presents 2.75 times higher probability of managing the prevention of labour risks (aOR = 2.75; 95% CI: 2.31–3.28) and 1.86 times higher probability that the workers participate in a passive way (aOR = 1.86; 95% CI: 1.60–2.17) than in those work centres where there are no such representatives. However, the presence of delegates had a negative impact on the active participation of workers (aOR = 0.59; 95% CI: 0.50–0.70) and was not related to the reduction of serious or fatal accidents and occupational diseases.

## 4. Discussion

In view of the growing concern in the European Union for the increase and effectiveness of unitary representation systems in occupational health and safety, the aim of this research was to study the impact of unitary prevention delegates in the Spanish working environment. The results obtained have found positive effects in their indirect dimensions as well as a positive direct impact on the work accidents themselves. In this sense, just as with unionised prevention delegates [38,39,40], unitary representation in occupational health and safety also has a positive impact on occupational risk prevention management, insofar as it has been accredited that, in work centres with unitary prevention delegates, prevention management indicators are higher than those in centres without representatives (indirect impact), which shows, above all that, in the period of economic crisis studied, prevention delegates are capable of putting pressure on management to comply with the LPRL. Again, coinciding with the studies focused on union representation [43,44], unit prevention delegates have been linked to those work centres with fewer minor occupational accidents (direct impact).

However, the results obtained show how prevention delegates are not capable of activating the active participation of workers in the management of occupational risks and reducing the probability of serious or fatal accidents and occupational diseases, demonstrating less effectiveness than union representation in occupational health, since previous studies have shown how union prevention delegates have been capable of activating participatory cultures [41,42]. In this sense, several studies [30,31,32] have found that only in those workplaces where workers are actively involved in identifying risks and designing and adopting prevention measures are better results achieved, i.e., the most serious damage to health is reduced. For this reason, we consider that the unitary prevention delegates have limited effectiveness, derived from their inability to activate the active participation of the workers, which is probably one of the factors that would explain why in this study they have not had relevant effects on the most serious damage to health. However, this statement could be studied in future studies.

The results obtained suggest that the limitations of the prevention delegates in Spain are due more to contextual factors associated with the interaction between the workers’ representatives and the management of the company and the interaction of the prevention delegates with the workers themselves, rather than to the configuration of the system of representation itself, whether unitary or trade union. Thus, the key factor would be the role adopted by the unitary prevention delegates in such interactions. In this sense, the studies by Hall et al. [63] show two possible roles or types of action by the prevention delegates: (1) a scientific-technical role in which the delegates adopt a technical vision by studying compliance with the concrete measures established by legislation, leaving little room for worker participation [64]; and (2) a politically active role in which representatives stop focusing on technical aspects and seek to solve holistic occupational safety and health problems, adopting a critical position on management and involving the active mobilisation of workers [63]. In this regard, qualitative studies by Ollé et al. [65] carried out in the Spanish workplace found that most unitary prevention delegates adopt a scientific-technical role as a defensive strategy, since company management tends to be hostile to worker participation. In fact, in the last European Survey of Companies on New and Emerging Risks (ESENER-3) conducted in 2019 [66], 89.6% of companies in Spain mainly carried out prevention actions to comply with legislation, with a similar situation in the rest of Europe, where the European average was 89.2%. Thus, in the face of a lack of capacity for action, prevention delegates adopted a defensive posture focusing on demanding compliance with the law [67], going to the Labour Inspectorate instead of mobilising and activating worker participation in the face of authoritarian management cultures [68]. 

In addition to the difficulties mentioned, there are other limitations arising from the weak position of the working class in the labour market (low structural power) [69], so that workers on temporary contracts or belonging to smaller subcontracting firms do not want to exercise their participation rights for fear of being fired and prefer to have their representatives handle occupational safety and health matters [65]. In fact, there is empirical evidence of the difficulties of participation by peripheral workers belonging to the weaker steps of the supply chain [70,71]. In this context, we can conclude that the system has entered a kind of spiral of constant deregulation of working conditions that has a negative impact on workers’ health (for example, in Table 5 we have seen how temporary workers and, above all, those belonging to subcontracting companies report more occupational accidents) and, in turn, prevents worker participation and the monitoring and control of their representatives, which feeds back into the spiral of deregulation. Thus, in addition to reversing this trend, it is necessary to activate a participatory culture in the workplace, developing knowledge and skills in safety and health through joint training systems for representatives and management that improve and clarify roles [70] and quality circles in occupational safety and health in which prevention delegates and workers participate [65]. 

In summary, the results obtained in this research and the debates held suggest that the consequences for the preventive system are, on the one hand, the legal bureaucratisation of risk prevention and, on the other hand, as a consequence of this legal technification, the difficulty of finding spaces for the real participation of workers in risk management, which in the end causes a kind of mirage of institutional security that does not have a real impact on the reduction of occupational accidents. Thus, for example, employers may only want to comply with preventive regulations to avoid being sanctioned and, to that end, they hire experts to carry out risk assessment or preventive planning, but they usually do not allow workers to participate in the management processes, i.e., management hires prevention services and, in doing so, they consider that they have already fulfilled their obligations [65]. Faced with the impossibility of participation, the prevention delegates focus their efforts on demanding compliance with the safety measures stipulated in the official documents in the labour inspection. For example, if the risk assessment detects that a machine does not have a safety guard and plans to establish protective systems on the machine, the conflict could arise when the employer tries to reduce the economic cost of the safety measure and simply provide personal protective equipment to its workers, but nevertheless, the effective measure would be to act on the machine itself by incorporating safety devices, but as they are more expensive or can cause delays in production, the employer rejects the measure, and it is there that the prevention delegates request compliance with the measure and, if necessary, report it to the labour inspectorate, which is responsible for checking whether or not the regulations have been complied with [35]. Thus, as can be seen, we are entering a spiral of legal compliance with preventive management, but with a clear lack of cultural activation of the different key actors (employers, representatives, workers and labour inspection) and, therefore, as has been mentioned, it is necessary to carry out awareness campaigns to improve the occupational safety and health systems in Spanish workplaces.

### Limitations

The study has some limitations, thus the results should be interpreted with caution. Firstly, although the survey controls for the degree of subjectivity of the responses when interviewing managers or specialist technicians responsible for managing prevention in the workplace, there may be subjective biases that direct their opinion towards what is considered to be socially accepted, since recognising that risks are not managed or that workers are not allowed to participate in such management and that accidents at work occur, may be controversial for the organisation’s reputation. Secondly, this is a cross-sectional study, which limits the inferences about the relationships between the variables and therefore prevents the establishment of their directionality. However, the results can be considered valid to the extent that they are controlled by variables related to labour precariousness (temporary contracts, subcontracted workers, night shift work, etc.) which previous studies have shown to be related to levels of management, participation and damage to health. Finally, it should be mentioned that the most recent survey carried out in Spain by the INSHT called “The preventive management of companies in Spain. Year 2016” [72], only has the indicators of preventive management, eliminating from the questionnaire all the questions related to worker participation and damage to occupational health. In fact, while the ENGE survey had 57 questions divided into nine blocks, in the current survey, there is only one block formed with nine questions. For this reason, to continue studying and consolidating the results obtained in the present research, it would be convenient to recover all the dimensions analysed in future preventive management surveys.

## 5. Conclusions

There is growing concern about the increasing systems of representation of non-unionised workers in occupational health and safety. Previous studies have shown that workplaces with unionised prevention delegates have better prevention management rates, greater worker participation and a reduction in occupational accidents. However, there is no comparable scientific evidence on the impact of unitary prevention delegates, and there is concern that their impact on occupational health and safety is lower. Therefore, the aim of this research was to study the direct and indirect impact of the unitary prevention delegates on the Spanish working environment. The results obtained have found how the unitary prevention delegates play a relevant role in promoting occupational health and safety management and in reducing minor occupational accidents, but there are some weaknesses related to the inability of the representatives to activate worker participation and, therefore, it is likely that they do not have a greater direct impact on serious or fatal occupational accidents and occupational diseases. Analysing the results and discussing them with previous studies, it seems that contextual factors (authoritarian corporate cultures and low structural power of workers in the labour market) influence more the levels of worker participation than the representation system itself. It would be interesting for future research to carry out qualitative studies, focusing on the controversies between systems of representation (trade union and unitary), direct worker participation and management roles, and to analyse how the occupational safety and health management system can constructively activate participatory cultures.

## Figures and Tables

**Table 1:** Distribution of unitary prevention delegates according to company size and activity sector.

	No Prevention Delegate No. (%) ^A^	With Prevention Delegate No. (%) ^A^	*p*-Value ^B^
Company size			0.000
<10	3053 (80.5)	740 (19.5)	
10–49	537 (51)	515 (49)	
50–249	50(24.4)	155 (75.6)	
250–499	3 (13.6)	19 (86.4)	
= >500	0 (0)	16 (100)	
Sector of activity			0.000
Agriculture	154 (75.9)	49 (24.1)	
Industry	496 (61.4)	312 (38.6)	
Construction	500 (67.8)	238 (32.2)	
Services	2496 (74.7)	847 (25.3)	

^A^ No., number of centres; (%), percentage of centres over the total of the corresponding subsample; ^B^ *p*-value, calculated from the Chi-square test with a 95% confidence level.

**Table 2:** Variables included in the study.

Dimensions	Indicator	Category
Independent variable	There are unitary prevention delegates in the workplace	1 = With prevention delegate 2 = No prevention delegate
Dependent variables		
Prevention management	It has been carried out in the workplace: 1. Risks evaluation 2. Prevention plan 3. Assignment of roles and responsibilities 4. Preventive planning 5. Priority setting and controls 6. Emergency plan 7. Definition of emergency measures 8. Emergency drills 9. Accident investigation	1 = Yes, it has been done 2 = No, it has been carried out or is planned
Participation of the workers	Passive participation: 1. Information. 2. Training. Active participation. Workers have been asked about: 3. Risks evaluation 4. Introduction of new technologies 5. Choice of work equipment and/or personal protective equipment 6. Prevention organisation 7. Organisation of training 8. Choice of third-party prevention service 9. Mutual Election	1 = Yes 2 = No
Damage to health	Health damage produced in the last two years: 1. Minor accidents. 2. Serious or fatal accidents. 3. Occupational diseases.	1 = Yes 2 = No
Adjustment Covariates		
Company type	Size of the company	1 ≤ 10 2 = 10–49 3 = 50–249 4 = 250–499 5 ≥ 500
	2. Activity sector	1 = Agriculture 2 = Industry 3 = Construction 4 = Services
Working conditions	Percentage according to own staff 1. Permanent employment 2. Temporary Percentage according to external staff: 3. Freelancers. 4. ETT 5. Outsourced. Percentage according to workday 6. Morning and afternoon 7. Continues tomorrow 8. Continuous afternoon 9. Continues at night 10. Morning and afternoon shifts 11. Shifts morning, afternoon and night Percentage according to sex: 12. Men 13. Women Percentage according to nationality 14. Nationals 15. Foreign Percentage according to age: 16. between 16 and 19 years 17. between 20 and 24 18. between 25 and 54 19. between 55 and 65 20. over 65 years	1-Above average. 2-Below average

**Table 3:** Prevalence and adjusted logistic regressions between the presence of unitary prevention delegates and preventive management indicators.

Indicator	No Prevention Delegate	With Prevention Delegate	cOR (95% CI) ^C^	aOR (95% CI) ^D^
	N (%) ^A^	N (%) ^B^		
Risks evaluation	2213 (71.8)	1131 (94.3)	6.45 (4.99–8.32) ^E^	5.96 (4.44–8.01) ^E^
Prevention plan	2088 (60.8)	1198 (88.5)	4.96 (4.14–5.95) ^E^	3.97 (3.26–4.83) ^E^
Assignment of roles and responsibilities	1042 (32.2)	739 (57.2)	2.82 (2.47–3.22) ^E^	2.15 (1.86–2.50) ^E^
Preventive planning	1802 (53.4)	1105 (81.5)	3.84 (3.30–4.48) ^E^	3.01 (2.55–3.56) ^E^
Priority setting and controls	1365 (41.7)	869 (65.9)	2.71 (2.37–3.09) ^E^	2.10 (1.82–2.44) ^E^
Emergency plan	948 (29.5)	659 (50.7)	2.46 (2.16–2.81) ^E^	2.06 (1.77–2.38) ^E^
Definition of emergency measures	1704 (51.2)	923 (69.1)	2.13 (1.86–2.44) ^E^	1.58 (1.36–1.83) ^E^
Emergency drills	762 (23.4)	599 (45.1)	2.69 (2.36–3.09) ^E^	2.16 (1.85–2.52) ^E^
Accident investigation	1055 (32.6)	719 (54.6)	4.48 (2.17–2.82) ^E^	1.50 (1.29–1.74) ^E^

^A^ N (%), number and percentage of workplaces without prevention delegates managing prevention; ^B^ N (%), number and percentage of workplaces with prevention delegates managing prevention; ^C^ oOR(CI 95%), crude odds ratio and 95% confidence interval, the reference category being the absence of prevention delegates; ^D^ aOR(CI 95%), odds ratio adjusted by the adjustment covariates in Table 2 and their corresponding 95% confidence interval, the reference category being the absence of prevention delegates; ^E^ significance level of the *p*-value association <0.001.

**Table 4:** Prevalence and logistical regressions adjusted between the presence of unitary prevention delegates and participatory management indicators.

Indicator	No Prevention Delegate	With Prevention Delegate	cOR (95% CI) ^C^	aOR (95% CI) ^D^
	N (%) ^A^	N (%) ^B^		
Passive participation				
Information	1838 (84.1)	998 (88.2)	1.41 (1.14–1.74) ^F^	1.38 (1.09–1.74) ^F^
Training	1915 (52.7)	1196 (82.9)	4.35 (3.74–5.06) ^E^	3.51 (2.97–4.15) ^E^
Active participation				
Risk assessment	1611 (64.0)	604 (46.3)	0.48 (0.42–0.55) ^E^	0.47 (0.40–0.55) ^E^
Introduction of new technologies	1140 (45.3)	392 (30.0)	0.52 (0.45–0.60) ^E^	0.57 (0.49–0.67) ^E^
Choice of work equipment and/or personal protective equipment	1273 (50.6)	561 (30.6)	0.74 (0.64–0.84) ^E^	0.71 (0.61–0.82) ^E^
Organisation of prevention	1125 (44.7)	403 (30.9)	0.55 (0.48–0.64) ^E^	0.61 (0.52–0.71) ^E^
Organisation of training	1296 (51.5)	435(33.3)	0.47 (0.41–0.54) ^E^	0.49 (0.42–0.57) ^E^
Choice of the external prevention service	590 (23.4)	194 (14.9)	0.57 (0.48–0.68) ^E^	0.71 (0.58–0.86) ^F^
Choice of the Mutual	81 (32.2)	235 (18.0)	0.46 (0.39–0.54) ^E^	0.57 (0.48–0.69) ^E^

^A^ N (%), number and percentage of workplaces without prevention delegates in which workers participate; ^B^ N (%), number and percentage of workplaces with prevention delegates in which workers participate; ^C^ oOR (CI 95%), crude odds ratio and 95% confidence interval, the reference category being the absence of prevention delegates; ^D^ aOR (IC 95%), odds ratio adjusted by the adjustment covariates in Table 2 and their corresponding 95% confidence interval, the reference category being the absence of prevention delegates; ^E^ significance level of *p*-value association <0.001; ^F^ significance level of *p*-value association <0.05.

**Table 5:** Adjusted logistical regressions between the presence of unitary prevention delegates and damage to occupational health.

	Minor Work Accidents ^A^		Serious or Fatal Accidents at Work ^A^		Occupational Diseases ^A^	
	aOR (95% CI) ^B^	*p*- Value	aOR (95% CI) ^B^	*p*- Value	aOR (95% CI) ^B^	*p*- Value
There is a unitary prevention delegate						
No	1 ^C^		1 ^C^		1 ^C^	
Yes	0.64 (0.53–0.76)	0.000	0.92 (0.51–1.66)	0.785	1.53 (0.84–2.76)	0.163
Socio-demographic adjustment variables						
Company size						
<10	1 ^C^		1 ^C^		1 ^C^	
10–49	2.46 (2.03–2.97)	0.000	5.27 (2.52–11.01)	0.000	5.05 (2.59–9.87)	0.000
50–249	4.08 (3.32–6.96)	0.000	12.18 (4.71–31.47)	0.000	8.56 (3.08–23.76)	0.000
250–499	8.66 (5.43–30.84)	0.001	115.6 (23.5–566.6)	0.000	6.20 (0.66–58.0)	0.110
=> 500	3.85 (1.16–12.73)	0.000	23.13 (2.96–180.4)	0.000	26.6 (3.94–180.5)	0.001
Activity sector						
Agriculture	1 ^C^		1 ^C^		1 ^C^	
Industry	0.98 (0.67–1.44)	0.925	0.81 (0.25–2.62)	0.728	1.12 (0.25–5.07)	0.881
Construction	1.90 (1.30–2.78)	0.001	1.04 (0.33–3.31)	0.943	1.13 (0.24–5.26)	0.877
Services	0.43 (0.30–0.62)	0.000	0.36 (0.11–1.14)	0.083	0.83 (0.20–5.26)	0.807
Percentage according to own staff ^D^						
Undefined	1.08 (0.83–1.41)	0.573	0.28 (0.10–0.74)	0.011	0.65 (0.27–1.56)	0.336
Temporary	1.35 (1.04–1.75)	0.027	0.34 (0.13–0.94)	0.037	0.53 (0.22–1.27)	0.155
Percentage according to external staff ^D^						
Freelancers	5.76 (1.99–16.65)	0.000	0.12 (0.01–5.32)	0.274	0.75 (0.08–7.38)	0.807
ETT	0.44 (0.22–0.88)	0.021	0.57 (0.12–2.75)	0.485	1.06 (0.40–2.78)	0.914
Subcontractor	2.91 (1.91–4.45)	0.001	3.07 (1.36–6.93	0.007	0.56 (0.22–1.44)	0.474
Percentage according to working day ^D^						
Morning and afternoon	1.13 (0.82–1.55)	0.459	0.79 (0.30–2.08)	0.627	2.60 (1.06–6.38)	0.038
Continues tomorrow	0.99 (0.73–1.34)	0.952	1.00 (0.40–2.46)	0.994	1.96 (0.92–4.18)	0.080
Continuous afternoon	1.90 (1.38–2.62)	0.000	1.16 (0.41–3.25)	0.784	1.80 (0.75–4.33)	0.190
Continues at night	0.77 (0.69–1.45)	0.363	2.00 (0.61–6.63)	0.255	0.73 (0.15–3.57)	0.700
Morning and afternoon shifts	1.00 (0.69–1.45)	0.997	2.23 (0.89–5.60)	0.088	2.83 (1.21–6.67)	0.017
Shifts morning, afternoon and night	5.26 (3.38–8.19)	0.000	0.76 (0.24–2.38)	0.632	6.01 (2.45–14.71)	0.000
Percentage according to sex ^D^						
Men	1.29 (1.11–1.51)	0.001	0.96 (0.56–1.63)	0.874	0.48 (0.29–0.82)	0.006
Women	0.83 (0.61–1.14)	0.384	0.25 (0.08–0.81)	0.021	0.29 (0.10–0.76)	0.013
Percentage according to nationality ^D^						
Nationals	0.97 (0.74–1.26)	0.804	1.51 (0.61–3.71)	0.373	0.56 (0.22–1.44)	0.228
Foreign	1.01 (0.78–1.32)	0.931	0.97 (0.42–2.26)	0.944	0.20 (0.07–0.61)	0.004
Percentage according to age ^D^						
Between 16 and 19 years	1.19 (0.84–1.68)	0.335	1.39 (0.55–3.50)	0.482	2.66 (1.22–5.80)	0.014
Between 20 and 24	1.48 (1.20–1.82)	0.000	1.47 (0.78–2.78)	0.235	3.03 (1.66–5.51)	0.000
Between 25 and 54	1.47 (1.18–1.83)	0.001	0.96 (0.49–1.91)	0.916	2.37 (1.10–5.11)	0.028
Between 55 and 65	1.71 (1.40–2.09)	0.000	1.93 (1.04–3.59)	0.037	1.11 (0.58–2.15)	0.746
Over 65 years	3.09 (1.98–4.80)	0.000	0.86 (0.23–3.28)	0.828	2.45 (0.72–8.30)	0.150
Chi squared	1062,416	0.000	182,161	0.000	132,867	0.000
Cox and Snell R^2^	0.194		0.036		0.027	
R^2^ Nagelkerke	0.303		0.275		0.198	
Population	5147		5147		5147	
Number of valid cases and percentage	4812 (93.5%)		4809 (93.4%)		4809 (93.4%)	

^A^ The reference categories are the absence of minor, serious or fatal accidents and occupational diseases in the workplace; ^B^ aOR (95% CI), adjusted odds ratio for all variables included in the model and their corresponding 95% confidence intervals; ^C^ the reference category in the independent variable and the size and sector of the enterprise; ^D^ the reference category is the enterprises that have a percentage below the average for each dimension.

## Appendix A

**Table A1:** Descriptive analysis of the sample.

Indicator	Category	No. (%)
Independent variable		
There are unitary prevention delegates in the workplace		
	With prevention delegate	1446 (28.1)
	No prevention delegate	3645 (70.8)
Dependent variables		
Prevention management		
Risks evaluation		
	Yes, it has been done	3344 (78.1)
	No it has been carried	937 (21.9)
Prevention plan		
	Yes, it has been done	3286 (68.6)
	No it has been carried	1501 (31.4)
Assignment of roles and responsibilities		
	Yes, it has been done	1781 (39.3)
	No it has been carried	2747 (60.7)
Preventive planning		
	Yes, it has been done	2907 (61.5)
	No it has been carried	1823 (38.5)
Priority setting and controls		
	Yes, it has been done	2234 (48.7)
	No it has been carried	2358 (51.3)
Emergency plan		
	Yes, it has been done	1607 (35.6)
	No it has been carried	2907 (64.4)
Definition of emergency measures		
	Yes, it has been done	2627 (56.3)
	No it has been carried	2037 (43.7)
Emergency drills		
	Yes, it has been done	1361 (29.7)
	No it has been carried	3223 (70.3)
Accident investigation		
	Yes, it has been done	1774 (39)
	No it has been carried	2.779 (61)
Participation of the workers		
Passive participation		
Information		
	Yes	2836 (85.5)
	No	481 (14.5)
Training		
	Yes	3111 (61.3)
	No	1966 (38.7)
Active participation		
Risks evaluation		
	Yes	2517 (58)
	No	1607 (42)
Introduction of new technologies		
	Yes	1532 (40.1)
	No	2292 (59.9)
Choice of work equipment and/or personal protective equipment		
	Yes	1834 (42.2)
	No	2515 (57.8)
Organisation of prevention		
	Yes	1528 (40)
	No	2293 (60)
Organisation of training		
	Yes	1731 (45.3)
	No	2092 (54.7)
Choice of third-party prevention service		
	Yes	784 (20.5)
	No	3039 (79.5)
Choice of the Mutual		
	Yes	316 (20.3)
	No	1242 (79.7)
Damage to health		
Minor accidents.		1064 (20.5)
Serious or fatal accidents.		69 (1.3)
Occupational diseases		70 (1.4)
Adjustment Covariates		
Size of the company		
	<10	3793 (74.7)
	10–49	1052 (20.6)
	50–249	205 (4.0)
	250–499	22 (0.4)
	≥500	16 (0.3)
Activity sector		
	Agriculture	203 (3.9)
	Industry	818 (15.9)
	Construction	739 (14.4)
	Services	3387 (65.8)
Working conditions (Above average)		
Percentage according to own staff		
	Permanent employment	3180 (61.8)
	Temporary	1491 (29.0)
Percentage according to external staff		
	Freelancers	24 (0.5)
	ETT	73 (1.4)
	Outsourced.	162 (3.1)
Percentage according to work day		
	Morning and afternoon	3542 (68.8)
	Continues tomorrow	1105 (21.5)
	Continuous afternoon	429 (8.3)
	Continues at night	97 (1.9)
	Morning and afternoon shifts	350 (6.8)
	Shifts morning, afternoon and night	156 (3.0)
Percentage according to sex:		
	Men	2549 (49.5)
	Women	2151 (41.8)
Percentage according to nationality		
	Nationals	3405 (66.2)
	Foreign	940 (18.3)
Percentage according to age		
	between 16 and 19 years	217 (4.2)
	between 20 and 24	1241 (24.1)
	between 25 and 54	3065 (59.6)
	between 55 and 65	1221 (23.7)
	over 65 years	129 (2.5)

No., number of centres; (%), percentage of centres over the total of the corresponding subsample.
